# Identification of Selective Small Molecule Inhibitors of the Nucleotide-Binding Oligomerization Domain 1 (NOD1) Signaling Pathway

**DOI:** 10.1371/journal.pone.0096737

**Published:** 2014-05-07

**Authors:** David J. Rickard, Clark A. Sehon, Viera Kasparcova, Lorena A. Kallal, Pamela A. Haile, Xin Zeng, Monica N. Montoute, Derek D. Poore, Hu Li, Zining Wu, Patrick M. Eidam, John G. Emery, Robert W. Marquis, Peter J. Gough, John Bertin

**Affiliations:** 1 Pattern Recognition Receptor Discovery Performance Unit, Immuno-Inflammation Therapeutic Area, GlaxoSmithKline, Collegeville, Pennsylvania, United States of America; 2 Biological Reagents and Assay Development, Platform Technology Sciences, GlaxoSmithKline, Collegeville, Pennsylvania, United States of America; 3 Screening and Compound Profiling, Platform Technology Sciences, GlaxoSmithKline, Collegeville, Pennsylvania, United States of America; McGill University, Canada

## Abstract

NOD1 is an intracellular pattern recognition receptor that recognizes diaminopimelic acid (DAP), a peptidoglycan component in gram negative bacteria. Upon ligand binding, NOD1 assembles with receptor-interacting protein (RIP)-2 kinase and initiates a signaling cascade leading to the production of pro-inflammatory cytokines. Increased NOD1 signaling has been associated with a variety of inflammatory disorders suggesting that small-molecule inhibitors of this signaling complex may have therapeutic utility. We utilized a cell-based screening approach with extensive selectivity profiling to search for small molecule inhibitors of the NOD1 signaling pathway. Via this process we identified three distinct chemical series, xanthines (SB711), quinazolininones (GSK223) and aminobenzothiazoles (GSK966) that selectively inhibited iE-DAP-stimulated IL-8 release via the NOD1 signaling pathway. All three of the newly identified compound series failed to block IL-8 secretion in cells following stimulation with ligands for TNF receptor, TLR2 or NOD2 and, in addition, none of the compound series directly inhibited RIP2 kinase activity. Our initial exploration of the structure-activity relationship and physicochemical properties of the three series directed our focus to the quinazolininone biarylsulfonamides (GSK223). Further investigation allowed for the identification of significantly more potent analogs with the largest boost in activity achieved by fluoro to chloro replacement on the central aryl ring. These results indicate that the NOD1 signaling pathway, similarly to activation of NOD2, is amenable to modulation by small molecules that do not target RIP2 kinase. These compounds should prove useful tools to investigate the importance of NOD1 activation in various inflammatory processes and have potential clinical utility in diseases driven by hyperactive NOD1 signaling.

## Introduction

NOD1 belongs to the NOD-like receptor (NLR) family of cytoplasmic pattern recognition receptors (PRRs), which together with cell surface PRRs including the Toll-like and C-type lectin receptors, sense pathogens and initiate innate immune responses [1]. These receptors not only trigger innate immunity in response to bacterial, fungal and viral infections through the recognition of conserved pathogen-derived molecules (mostly nucleic acids and cell wall constituents) but are also activated by host-derived “danger” signals including factors release by cells undergoing necrosis [2]. Like the other PRRs, NOD1 is expressed by cell types well positioned for surveying and responding to invading micro-organisms, including macrophages, dendritic cells, neutrophils and epithelial cells of mucosal barrier tissues [3]. Activation of NOD1 in antigen presenting cells also serves to co-ordinate innate immune cell activation with an optimal adaptive immune response in T and B lymphocytes [4]. However, in addition to classical innate immune cell types, NOD1 is also present in numerous non-immune cells and tissues including vascular endothelium and mesenchymal/connective tissue cells suggesting a broader role in inflammation.

NOD1 activation by diaminopimelic acid (DAP), a peptidoglycan component of gram negative bacteria, stimulates release of inflammatory cytokines and chemokines via NF-κB and MAPK signaling pathways [5], [6]. Ligand binding to the C-terminal leucine rich repeat (LRR) domain of NOD1 is believed to induce a conformational change followed by ATP/nucleotide binding and oligomerization mediated by the central NACHT domain. These structural changes enable the formation of a polyubiquitinated signaling complex, the “nodosome”, via a homotypic CARD-CARD interaction with RIP2 serine/threonine kinase and subsequent recruitment of TAB1/TAK1 [7]. Although the immediate activating ligand for NOD1 has remained enigmatic, as for most other NLRs, recent data suggests that DAP fragments themselves can bind and induce a conformational change in purified NOD1 protein [8], [9]. In addition to inflammatory cytokine production induced via NF-κB and MAPKs, NOD1 activation in intestinal epithelial cells stimulates IFNβ secretion through a RIP2 and IRF7-dependent type I interferon pathway [10].

Numerous examples are now known where mutations in genes encoding PRRs are responsible for, or are associated with, autoimmune and inflammatory disease, thus emphasizing their prominent role as initiators of the innate inflammatory cascade. Polymorphisms in the *CARD4* gene encoding NOD1, are associated with susceptibility to inflammatory bowel disease, asthma and eczema [11]–[13]. Although the mechanisms underlying the disease association of NOD1 polymorphisms remain unknown, altered ligand dependency due to alternative splicing in the LRR domain has been proposed. In line with the wider tissue distribution of NOD1 compared to most other PRRs including NOD2, recent evidence indicates roles for NOD1 in diverse disorders including type 2 diabetes associated adipose tissue inflammation and in pathogen-induced inflammation in vascular endothelium [14]–[16]. Consequently, not only does NOD1 inhibition represent a novel anti-inflammatory approach with potentially broad therapeutic application but selective inhibitors of NOD1 signaling would allow the contribution of NOD1 activation to disease pathology to be evaluated.

Small molecule selective NOD1 inhibitors have previously been identified from a National Institutes of Health (NIH) compound collection using a cell-based screening assay for inhibitors of NOD1-stimulated NF-κB reporter gene activity [17], [18]. Such phenotypic cell-based screening is a feasible alternative strategy to a biochemical screen particularly for target proteins like NOD1 which have proven notoriously difficult to express and purify in a functional form in quantities sufficient for interrogating large compound libraries. Therefore, we performed a cellular high-throughput screen of the GSK collection (∼2 million compounds) for selective NOD1 pathway inhibitors that blocked cytokine production stimulated by DAP, but not by agonists of other pro-inflammatory pathways. This was analogous to a screening strategy we recently employed to identify selective inhibitors of MDP-stimulated NOD2 signaling [19]. We describe the identification and initial characterization of three structurally distinct chemical series of NOD1 pathway inhibitors, two of which are structurally different from those previously identified. Compared to the previously reported inhibitors [17], [18], each of the series we identified was demonstrated to inhibit both NOD1-stimulated NF-κB and MAPK signaling, exhibit selectivity against additional inflammatory pathways and one series was optimized to achieve significantly higher NOD1 inhibitory activity. These compounds therefore represent valuable tools to elucidate the importance of NOD1 activation in various inflammatory processes through its pharmacological inhibition.

## Materials and Methods

### Ethics Statement

The human peripheral blood that was used in these studies as a source of primary monocytes was obtained from normal healthy volunteers with informed written consent and procedures specifically approved by the GSK institutional review board.

Mice were used in these studies as a source of bone marrow-derived macrophages. All procedures were performed in accordance with a protocol (AUP0229/PA0644) that was specifically approved by the GSK Institutional Animal Care and Use Committee. The procedures employed in the protocol met or exceeded the standards of the Association for the Assessment and Accreditation of Laboratory Animal Care, International (AAALAC), the United States Department of Health and Human Services and all local and federal animal welfare laws.

### Reagents

The HEK293-hNOD1 (293/hNOD1), -hNOD2 and -hTLR2 cell lines were purchased and used under license from Cayla-InvivoGen. HCT116 and HT-29 colorectal carcinoma and adenocarcinoma cells, respectively, were obtained from American Type Culture Collection (ATCC). The NOD activators iE-DAP, Tri-DAP (L-Ala-γ-D-Glu-mesoDAP), muramyldipeptide (MDP), the TLR2/6 ligand Pam_2_CSK4, and Blasticidin S antibiotic for maintenance culture of stable cell lines were obtained from InvivoGen. The synthetic tetra-DAP peptide FK156 was synthesized by Biodura. The small molecule RIP2 and IKK inhibitors used as control compounds in these studies were developed at GSK and exhibit activities to their target human kinase of RIP2: IC_50_ = 3 nM, IKK2: IC_50_ = 1 nM, respectively.

### Compound Synthesis

The procedures used for compound synthesis and confirmation of compound structure are provided in Methods S1.

Physicochemical analyses of compounds were completed at GSK. These procedures included solubility determination by precipitation of 10 mM DMSO stock concentration to 5% DMSO phosphate buffered saline pH 7.4, with quantification by chemiluminescent nitrogen detection (CLND solubility), permeability across an artificial membrane, chromatographic hydrophobicity index (Chromlog D, a measure of lipophilicity), polar surface area (PSA, the surface map of a molecule describing the polar versus non-polar area), immobilized artificial membrane (IAM) binding, human serum albumin (HSA) binding and α1-acid glycoprotein (AGP) binding.

### Screening Assays

Inhibitors of iE-DAP-stimulated IL-8 release were screened in a 1536-well assay in which 293/hNOD1 cells (5000 cells/well) in DMEM high glucose medium supplemented with 0.1% FBS and 10 µg/mL Blasticidin S were stimulated with 60 ng/mL (200 nM) iE-DAP (EC_80_) in the presence of 10 µM compounds. After 24 hours the amount of IL-8 secreted into the medium was determined by homogeneous time resolved fluorescence (HTRF) assay by the direct addition of a 1∶1 mix of europium cryptate and XL665-conjugated anti-IL-8 antibodies according to the manufacturer’s instructions (Cisbio). Fluorescence was measured after 2 hours in a ViewLux imager using UMB-AMC excitation and 618–671 nm emission filters (Perkin Elmer). Subsequent assays for IC_50_ determination were performed in 384-well plates with 293/hNOD1, 293/hNOD2 or 293/hTLR2 cells (5000 cells/well) in the above medium and stimulated with the EC_80_ concentration of the appropriate activator/ligand (60 ng/mL iE-DAP, 30 ng/mL MDP, or 3–10 ng/mL Pam_2_CSK4). Inhibition of TNFR1-induced IL-8 release was performed with 293/hNOD1 cells stimulated with 20 ng/mL (∼EC_95_) rhTNFα.

Direct effects of compounds on RIP2 kinase activity was determined by autophosphorylation of full length human FLAG-6His tagged RIP2 expressed and purified from baculovirus. Enzyme was pre-incubated with compounds in assay buffer (50 mM HEPES pH 7.5, 25 mM MgCl_2_, 0.05%[w/v] CHAPS, 1 mM DTT and 0.05 mg/mL BSA) in 384-well plates, and then incubated with 10 µM ATP for 2 hours at room temperature. The amount of ADP generated was measured using the ADP-Glo universal ADP detection assay for kinases (Promega) and luminescence quantified on a ViewLux imager.

For the three chemical series subsequently progressed, the corresponding original hit compound was profiled for inhibition of kinase activity against a panel of 300 human kinases (Reaction Biology Corp). The compounds were tested in duplicate at 1 µM with a 20-minute pre-incubation with enzyme followed by addition of 10 µM ATP.

### Profiling Assays in Intestinal Epithelial Cells

Compound activity on endogenous NOD1 and NOD2 receptors was determined by inhibition of Tri-DAP or MDP-stimulated IL-8 release using HCT116 human colon carcinoma cells, maintained in DMEM high glucose medium supplemented with 25 mM HEPES and 10% FBS. For assay, cells were seeded into 96 well plates (90,000 cells/well) in DMEM containing 1% FBS and pre-incubated with compounds for 1 hour before addition of either 25 µg/mL Tri-DAP or 1 µg/mL MDP. The level of IL-8 released into medium after 24-hour incubation was determined by HTRF assay (Cisbio) as described above and fluorescence measured on an Envision model 2102 multilabel plate reader (Perkin Elmer). The activity of compounds against NOD1-induced type I interferon response was determined by inhibition of Tri-DAP stimulated IP-10 secretion in HT-29 cells, maintained in the same medium as for HCT116 cells. The HT-29 cells were seeded into 96 well plates (60,000 cells/well) in DMEM with 1% FBS, pre-incubated with compounds for 1 hour and stimulated for 24 hours with 50 µg/mL Tri-DAP. The concentration of IL-8 released into the medium was measured by HTRF as above, IP-10 was measured using the IP-10/CXCL10 Quantikine ELISA kit (R&D Systems), and IFNβ was measured using the MA6000 human IFN-β tissue culture kit (Meso Scale Discovery).

### Human Monocyte Isolation and Culture

Monocytes were purified from heparinized human whole blood by centrifugation on Ficoll (Histopaque 1077) followed by Percoll gradients. Monocyte purity was >95% by morphological assessment of Wright’s stained cytospins. Cells were seeded at 100,000 per well in 96-well plates in RPMI containing 10%HI-FBS, pre-incubated for 1 hour with compounds, and then stimulated with either 25 µg/mL Tri-DAP or 1 µg/mL MDP. The concentration of IL-8 in medium samples collected after 24-hour treatment was determined by HTRF whereas IL-1β, IL-6 and TNFα were measured using the MS6000 human pro-inflammatory-4 II tissue culture kit (Meso Scale Discovery).

### Murine Bone Marrow Derived Macrophage Culture

C57BL/6 mice were obtained from the Jackson Laboratories. Bone marrow was flushed from the femurs and tibiae of adult (∼3 month old) mice with serum-free DMEM medium using a 23-gauge needle and syringe. A fine cell suspension was obtained by gentle aspiration and passage through a 0.45 µm cell strainer. After centrifugation and re-suspending in DMEM supplemented with 10%HI-FBS, the mononuclear cells obtained from each animal were plated into the central 60 wells of a 96-well plate. After 24 hours, the medium and non-adherent cells were removed and replaced with DMEM with 10%HI-FBS containing 10 ng/mL M-CSF and the cells cultured for 1 week with two additional medium changes. After this time the medium was aspirated and the cells pre-treated with compounds for 1 hour and then stimulated for 24 hours with either 25 µg/mL Tri-DAP or FK156. Each treatment was performed in triplicate wells. The concentration of murine KC (CXCL1) together with 6 other cytokines/chemokines present in conditioned medium was then determined using the murine pro-inflammatory 7-plex assay (Meso Scale Discovery).

### Western Blotting

Confluent cultures of 293/hNOD1 and -hNOD2 cells seeded in 24-well plates were serum-starved over-night, pre-incubated for 1 hour with compound and then stimulated for 1 hour with either 50 µg/mL Tri-DAP or 25 µg/mL MDP, respectively. Whole cell lysates were prepared in 1X cell lysis buffer (Cell Signaling Technology) supplemented with additional protease, aminopeptidase and phosphatase inhibitors (protease inhibitor cocktail set III, EMD Biosciences; phosphatase inhibitor sets II and III, Sigma-Aldrich). The levels of phosphorylated and total p38, JNK, ERK1/2 and total IκBα were determined by immunoblotting of ∼20 µg protein per sample using the following primary antibodies: phospho-p38 MAPK (12F8, #4631), p38 MAPK (#9212), phospho-JNK (#9251), JNK (#9252) and IκBα (#9242) all purchased from Cell Signaling Technology, and phospho-ERK1/2 (sc-7383) and ERK1/2 (sc-94) antibodies from Santa Cruz Biotechnology.

## Results and Discussion

### Cell-based Approach for Identification of Selective NOD1 Pathway Inhibitors

**Figure 1:**
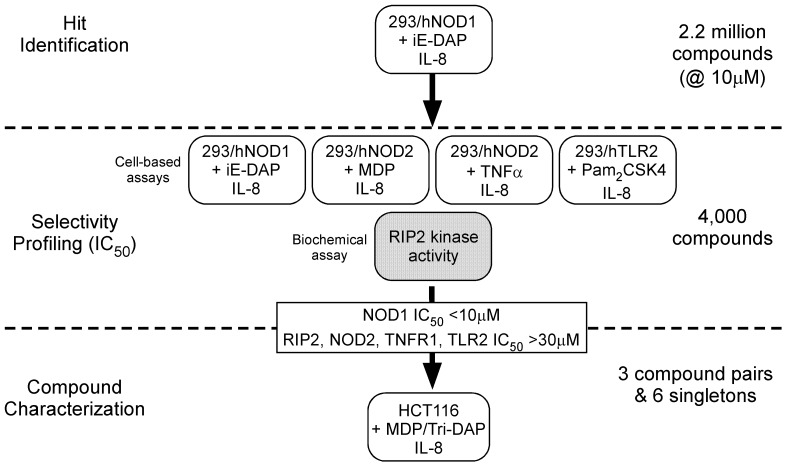
Scheme used to identify selective inhibitors of NOD1 stimulated IL-8 release in cells. In the primary HTS compounds that prevented iE-DAP induced cytokine in 293/hNOD1 cells were determined, followed by counter-screens to eliminate those compounds that also inhibited IL-8 induced via activation of NOD2, TNFR1 or TLR2, as well as compounds which directly inhibited RIP2 kinase activity. The activity of selective NOD1 inhibitors was then confirmed in HCT116 intestinal epithelial cells which express NOD1/2 endogenously, stimulated with either Tri-DAP or MDP.

**Figure 2:**
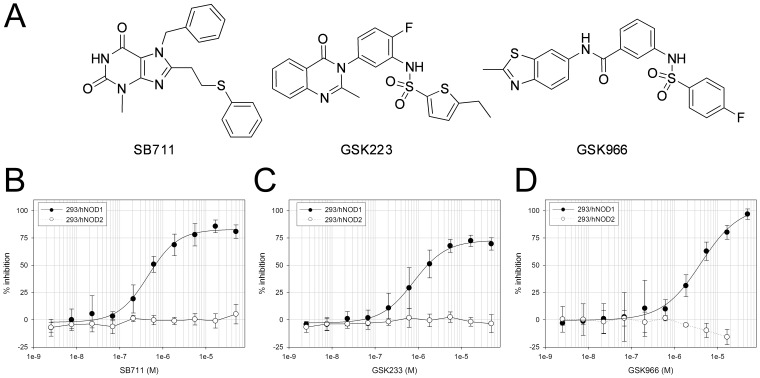
Activity of three structurally distinct selective NOD1 pathway inhibitors. (A) Chemical structure of the original hits in each series. (B–D) Concentration response curves for inhibition of IL-8 release by 293/hNOD1 and 293/hNOD2 stable cell lines stimulated with iE-DAP or MDP, respectively, and pre-incubated with the xanthine SB711 (B), quinazolininone GSK223 (C) or aminobenzothiazole GSK966 (D) over the concentration range 2.5 nM –50 µM. Data are the mean ± SD from at least 5 (293/hNOD1– iE-DAP) and from 2 (293/hNOD2– MDP) separate assays for each compound.

A high-throughput screen of about 2.2 million compounds was conducted for inhibitors of the NOD1 signaling pathway using suppression of iE-DAP stimulated IL-8 secretion in HEK293 cells stably expressing human NOD1 (293/hNOD1 cells). Confirmed hit compounds were then profiled in full dose-response in this assay as well as in four selectivity assays, to identify and de-select those compounds that also inhibited signaling through either NOD2 (MDP stimulated IL-8 secretion in 293/hNOD2 cells), TLR2 (Pam_2_CSK4 stimulated IL-8 secretion in 293/hTLR2 cells), TNFR1 (TNFα stimulated IL-8 secretion in 293/hNOD1 cells) or that directly inhibited RIP2 kinase activity (auto-phosphorylation of purified full-length RIP2). These assays were chosen to assess the selectivity of hits because the signaling pathways activated by these receptors converge at the level of the TAB/TAK1/IKK complex. Hence, we reasoned that hit compounds also inhibiting in one or more of the selectivity assays target either shared components downstream of TAB/TAK1 or receptor-proximal upstream components unique to each pathway and not involved in NOD1 signaling. Using this strategy, outlined in Figure 1, three compound pairs and six singletons were found that only blocked iE-DAP stimulated IL-8 release (IC_50_≤10 µM) and were essentially inactive in all of the other assays (IC_50_≥30 µM). Following preliminary analogue profiling for NOD1 inhibition and cellular toxicity, three chemical series all exhibiting selectivity for NOD1 were chosen for further development: the xanthines and two biarylsulphonamide series with quinazolininone or benzothiazole moieties (Figure 2A). The original hits in the xanthine, quinazolininone and benzothiazole series – SB711, GSK223 and GSK966– had NOD1 inhibitory activity in the 293/hNOD1 cell assay of IC_50_ = 0.6, 1.0 and 3.5 µM, respectively (Figure 2B–D, Table 1). Interestingly, the xanthine compound SB711 is structurally related to the purine-2,6-dione probe that was independently identified by a cell-based screen for inhibitors of NOD1-stimulated NF-κB signaling (PubChem ML-146, Magnuson et al, 2010, High throughput screening assays for NOD1 inhibitors – Probe 2. Probe Reports from the NIH Molecular Libraries Program [Internet]; National Center for Biotechnology Information (US): Bethesda, MD, 2010. PubMed PMID: 21433392, available from http://www.ncbi.nlm.nih.gov/books/NBK50701/).

**Table 1:** Activity of NOD1 selective compounds and inhibitors of IKK and RIP2 in cell-based assays used for hit identification and triage.

	IC50 (µM)							
		NOD1/2 selectivity assays				Specificity assays		
Compound	Monocyte Tri-DAP	293/hNOD1 iE-DAP	293/hNOD2 MDP	HCT116 Tri-DAP	HCT116 MDP	293/hNOD1 TNFα	293/hTLR2 Pam2CSK4	RIPK2 activity
GSK223	1.0	1.0	>50	0.4	>50	>50	>50	>50
CMPD **22**	0.06	0.06	>50	0.03	>15	>50	>50	>50
GSK996	2.0	3.5	>50	0.7	>50	>50	>50	>100
CMPD **16**	0.2	0.4	>30	0.06	ND	>30	>50	>50
SB711	3.2	0.6	>50	0.9	>50	>50	>50	>100
RIP2 inh	0.02	0.05	0.03	0.03	ND	>50	>10	0.003
IKK inh	>5	0.25	0.3	0.2	0.07	0.2	14.5	3.2

IC_50_ values are given in micromolar. For each of the cell-based assays, the concentration of IL-8 released into medium was the end-point measured. For the RIPK2 biochemical assay, the level of RIPK2 autophosphorylation was measured. Cell-based assays included are:- Monocyte = human primary peripheral blood monocytes; 293/hNOD1 and 293/hNOD2 = HEK293 cell lines stably expressing full-length human NOD1 or NOD2, respectively; HCT116 = human colon carcinoma cells.

Since each of the three hit compounds possessed structural motifs that could potentially act as kinase activation loop/hinge binders, they were profiled for inhibitory activity against 300 kinases. Promisingly, GSK996 and SB711 each weakly inhibited only a single kinase (GSK996 decreased CAMK1 activity by 55%, SB711 decreased EPHB2 activity by 51%) while GSK223 did not inhibit any kinase >50% (Figure S1). These results, together with the lack of inhibition of RIP2 kinase (above), indicate that the hit compounds are unlikely to block NOD1 signaling by inhibiting either a known, or an as yet unidentified, kinase that is unique to the NOD1 pathway but not required by any of the other signaling pathways used in our selectivity screening.

**Figure 3:**
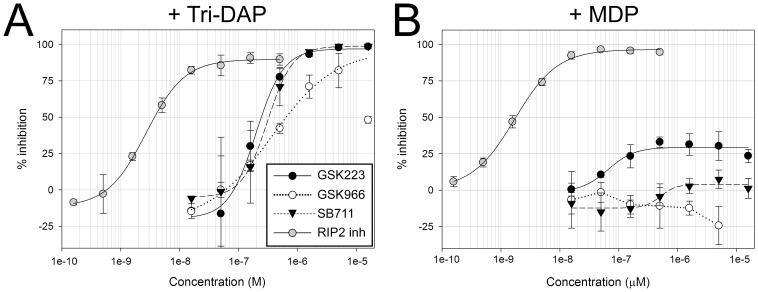
Activity of the three selective NOD1 pathway inhibitor compounds in cells with endogenous NOD1. Concentration response curves for the aminobenzothiazole GSK966, quinazolininone GSK223 and xanthine SB711 for inhibition of IL-8 secretion by HCT116 colon carcinoma cells pre-incubated for 1 hour with compounds (16 nM–16 µM) and then stimulated with 25 µg/mL Tri-DAP (A) or 1 µg/mL MDP (B). The concentration of IL-8 in medium was determined by HTRF assay after 24 hour treatment. Data are mean ± SD from a single experiment, repeated at least 3 times in Tri-DAP stimulated cells with similar results.

The three hit compounds also exhibited selective inhibition of NOD1 responses, with minimal or no inhibition of MDP-stimulated NOD2 signaling, in HCT116 colon carcinoma cells which endogenously express both NOD proteins at functional levels. Thus, increasing concentrations of SB711, GSK223 and GSK966 lead to the complete inhibition of Tri-DAP -stimulated IL-8 secretion in these cells, with IC_50_ values comparable to those in 293/hNOD1 stable cells (Figure 3, Table 1). Tri-DAP was the preferred NOD1 agonist in HCT116 cells owing to its greater induction of IL-8 compared to iE-DAP in these cells (data not shown). In contrast, none of the compounds appreciably inhibited MDP-stimulated IL-8 release in HCT116 cells, with GSK223 producing the greatest inhibition that reached only 30% even at high concentrations (Figure 3B).

### Inhibition of NOD1 Induced NF-κB and MAPK Pathways

**Figure 4:**
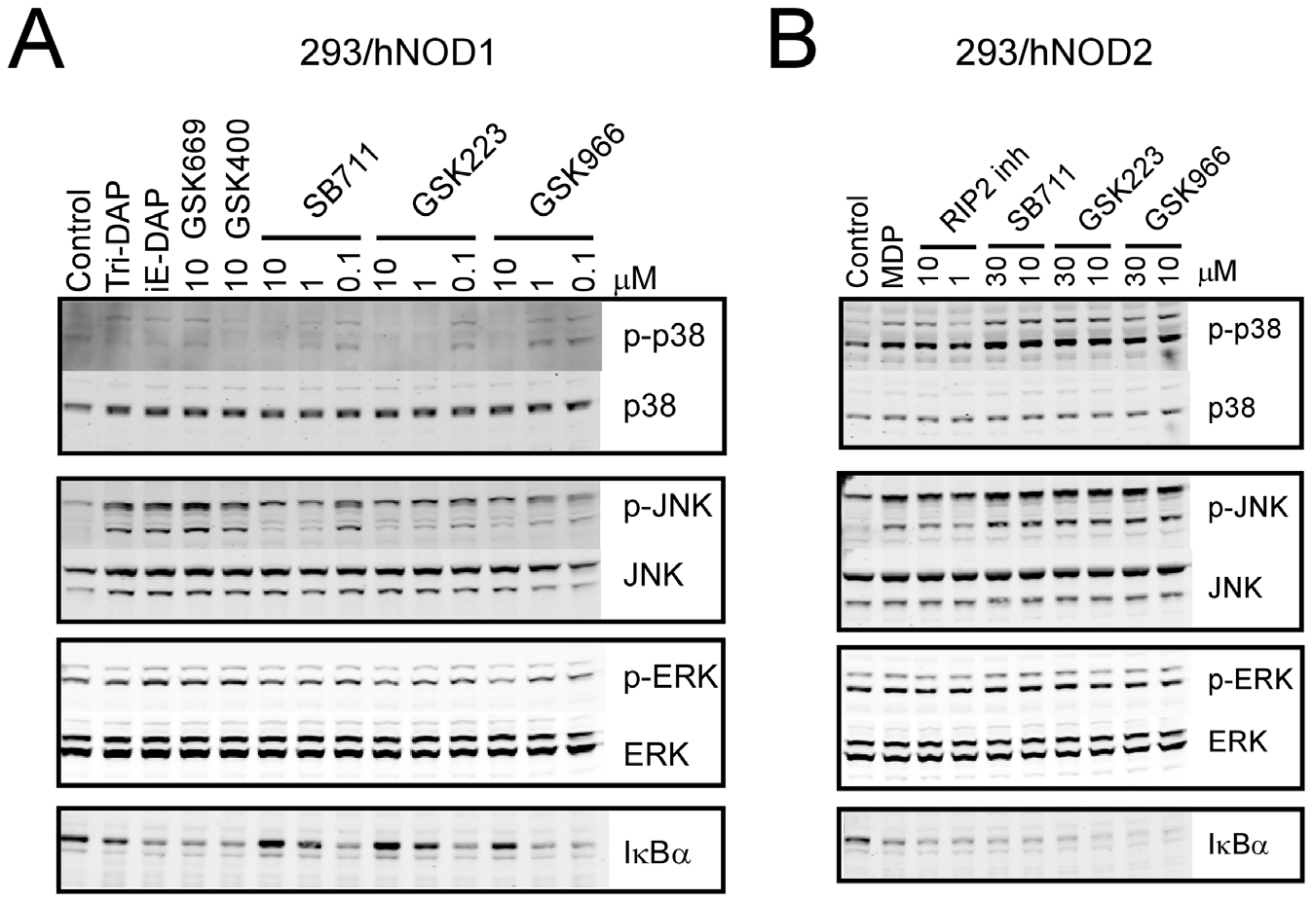
NOD1 pathway inhibitors block NF-κB and MAPK pathways. Serum-starved 293/hNOD1 (A) or 293/hNOD2 (B) stable cells were pre-incubated with compounds at the indicated concentrations prior to stimulation for 1 hour with either Tri-DAP (50 µg/mL) or MDP (25 µg/mL), respectively. Compounds included the three selective NOD1 pathway inhibitors or previously identified inhibitors of NOD2 signaling (GSK669 and GSK400). Levels of total IκBα, as well as total and phosphorylated MAPKs (p38, JNK and ERK1/2) were determined by immunoblotting of whole cell lysates. A RIP2 inhibitor was used as a positive control with 293/hNOD2 cells in which the NOD1 pathway inhibitors had no effect on MDP responses. Results shown are representative of two separate experiments performed in both cell lines.

NOD1 and NOD2 stimulate pro-inflammatory cytokine and chemokine release primarily via the activation of NF-κB and MAPK pathways. The ability of the original hit compounds from each series to inhibit both pathways in a NOD1-selective manner was investigated by western blotting for total IκBα and phosphorylated p38, JNK and ERK1/2 in 293/hNOD1 and -hNOD2 stable cell lines stimulated with Tri-DAP or MDP, respectively. As shown in Figure 4, NOD1 stimulation resulted in increased phospho-JNK, decreased IκBα, a barely discernible increase in phospho-p38 and no change in phospho-ERK. Each of the compounds antagonized the Tri-DAP induced increase in p-JNK levels and reduction in IκBα in 293/hNOD1 cells in a concentration-dependent manner from 0.1–10 µM (Figure 4A) but had no effect on the same responses to MDP in 293/hNOD2 cells even at higher concentration (30 µM) (Figure 4B). Since there was virtually no change in either phospho-p38 and phospho-ERK after NOD1 stimulation it was difficult to observe an inhibitory effect of the compounds on these components. In contrast, two inhibitors selective for MDP-stimulated NOD2 signaling, GSK669 and GSK400 [19], did not affect the phospho-JNK and IκBα responses to Tri-DAP (Figure 4A) whereas the RIP2 inhibitor blocked the responses of both Tri-DAP and MDP, although only the effects of the RIP2 inhibitor in MDP stimulated 293/hNOD2 cells are shown here since the NOD1 compounds were without effect in these cells (Figure 4B). The inhibitory activity towards Tri-DAP stimulated MAPK pathways was retained by more active compounds developed by subsequent lead optimization. For example, exemplar compounds from both quinazolininone and aminobenzothiazole series (compounds **9** and **16**, respectively, see below) reduced both phospho-JNK and the weaker phospho-p38 levels while levels of IκBα were increased following treatment of 293/hNOD1 cells with 10 µM or more of compound (Figure S2).

### Optimization and SAR

**Figure 5:**
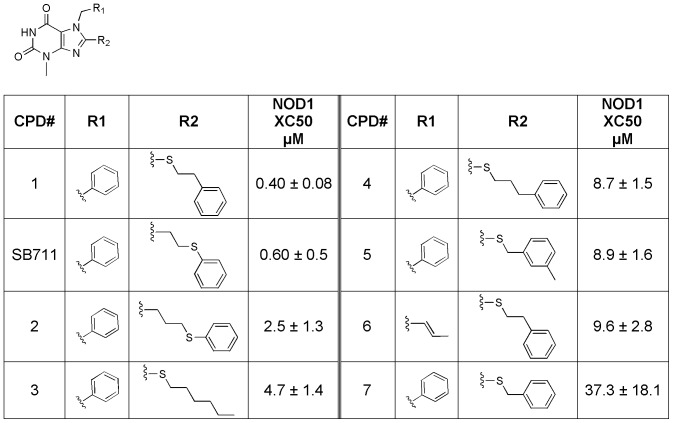
Structure-activity relationship for xanthine analogs (SB711 and compounds 1–7) in the iE-DAP-stimulated IL-8 production assay in 293/hNOD1 cells.

After hit confirmation and validation, further exploration of each series was initiated. Pharmacophore mapping, collection mining, and screening for related analogs allowed for further expansion of the SAR within each series. For the xanthine core (SB711, see Figure 5), both extending the tethered phenyl ring (compounds **2** and **4**) and shortening (compound **7**) are tolerated, but shortening the linker gave the greatest loss of activity. The optimal linker length for the 8-position appears to be three carbons (compound **1** vs SB711). The relative position of the sulfur in the linker does not appear to influence activity (compound **1** vs SB711 and compound **2** vs **4**). Also, the appended phenyl ring can be removed without a significant loss of activity (compound **3** vs **4**). Interestingly, and as mentioned above, this series is very similar to a series identified by Reed and co-workers (probe ML146, Magnuson et al, 2010, URL provided above) with compound **6** being most analogous. Indeed SB711 exhibits comparable activity to ML146 in NOD1-dependent cellular assays (ML146 IC_50_ = 1.5 µM in NOD1 agonist-stimulated NF-κB reporter assay in HEK293T cells). It should also be noted that the SAR for SB711 we observed is in agreement with that reported for ML146 in that minor changes in linker length at the 8-position and phenyl substitution (at position R1 in Figure 5) had only minor affects on activity.

**Figure 6:**
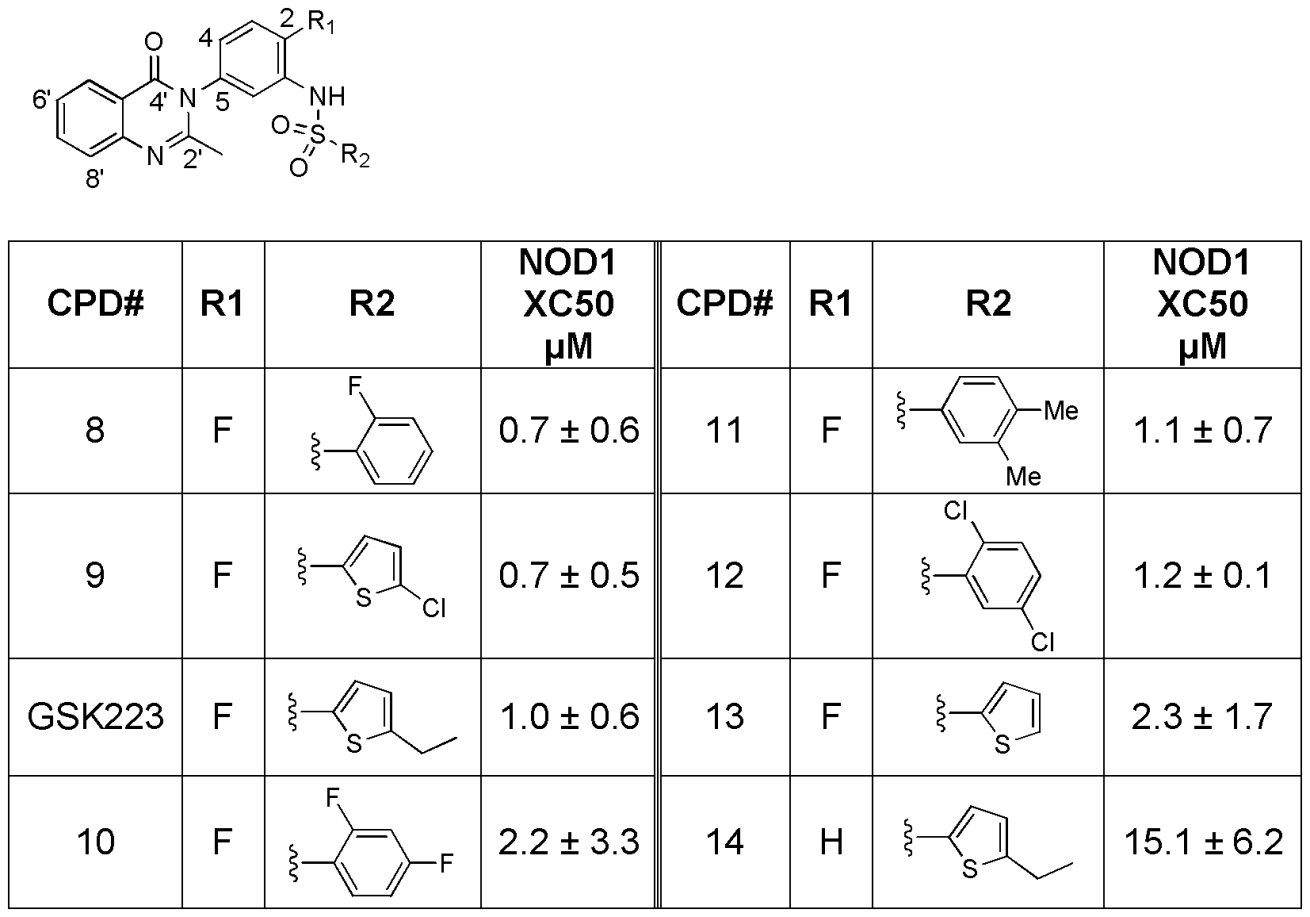
Structure-activity relationship for quinazolininone analogs (GSK223 and compounds 8–14) in the iE-DAP stimulated IL-8 production assay in 293/hNOD1 cells.

The quinazolininone biarylsulfonamides (GSK223, see Figure 6) showed the most promising activity from screening. Initial collection mining focused on variation of the aryl ring derived from the sulfonyl chloride while maintaining the remaining core. A wide range of substitutions were tolerated on both the thiophene and aryl inputs with the most active analogs possessing either ortho-fluoro aryl or chlorothiophene (compounds **8** and **9**, respectively, both with 293/hNOD1 IC_50_ = 0.7 µM). Interestingly, removal of the fluoro from the 2-position on the core phenyl (position R1 in Figure 6) results in a fairly dramatic drop in activity (GSK223 293/hNOD1 IC_50_ = 1.0 µM vs compound **14** = 15.1 µM).

**Figure 7:**
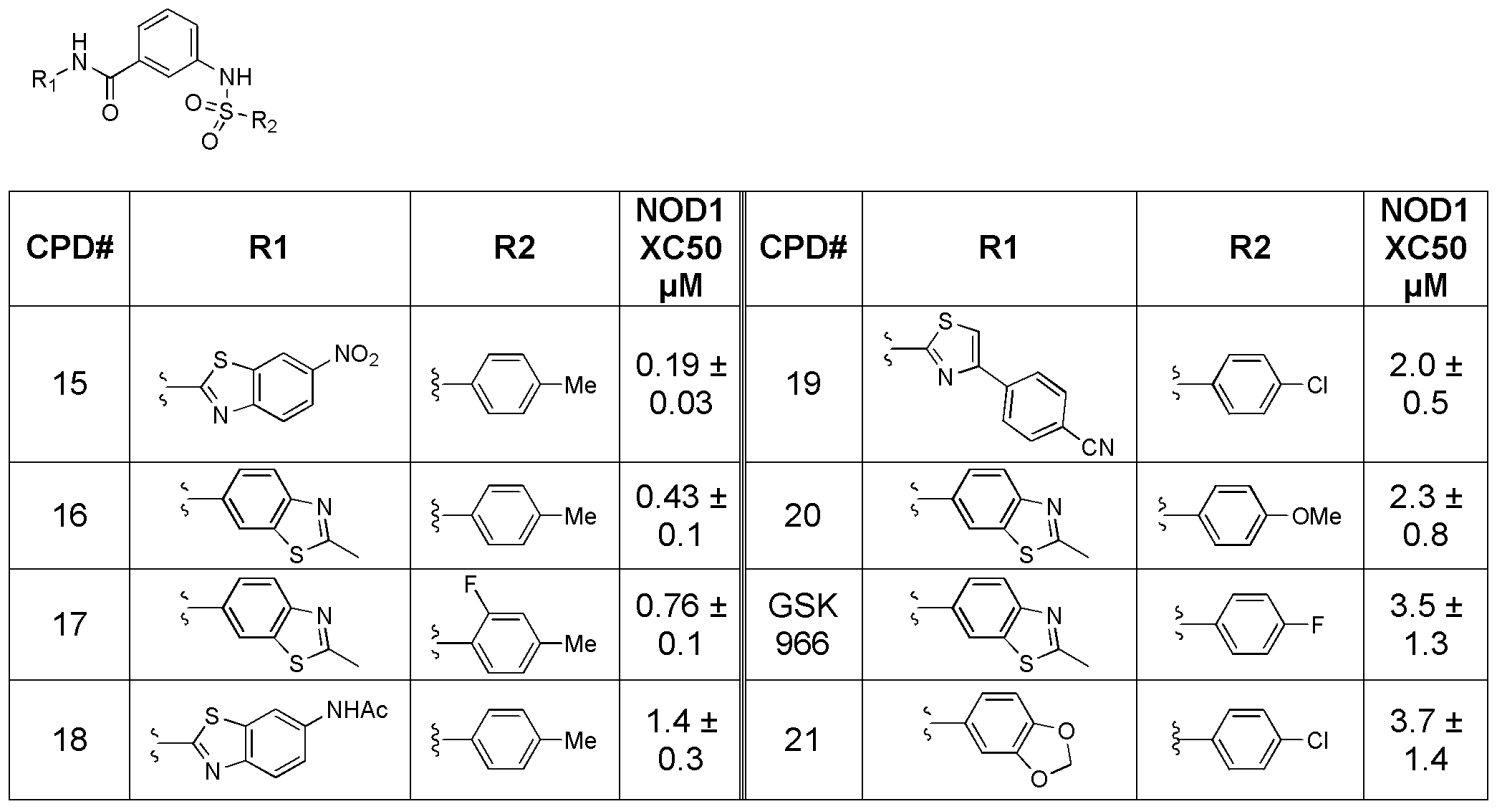
Structure-activity relationship for aminobenzothiazole analogs (GSK966 and compounds 15–21) in the iE-DAP stimulated IL-8 production assay in 293/hNOD1 cells.

For the aminobenzothiazole biarylsulfonamides (GSK966, see Figure 7), hit expansion identified compounds which were significantly more active than the original hit (GSK966 293/hNOD1 IC_50_ = 3.5, compound **15** = 0.2 µM). In addition, a wide array of substitutions were tolerated at both the amide linkage (compounds **15**, **18**, **19** and **21**) as well as the aryl ring derived from the sulfonyl chloride (compounds **16**, **17**, **19** and **20**).

**Figure 8:**
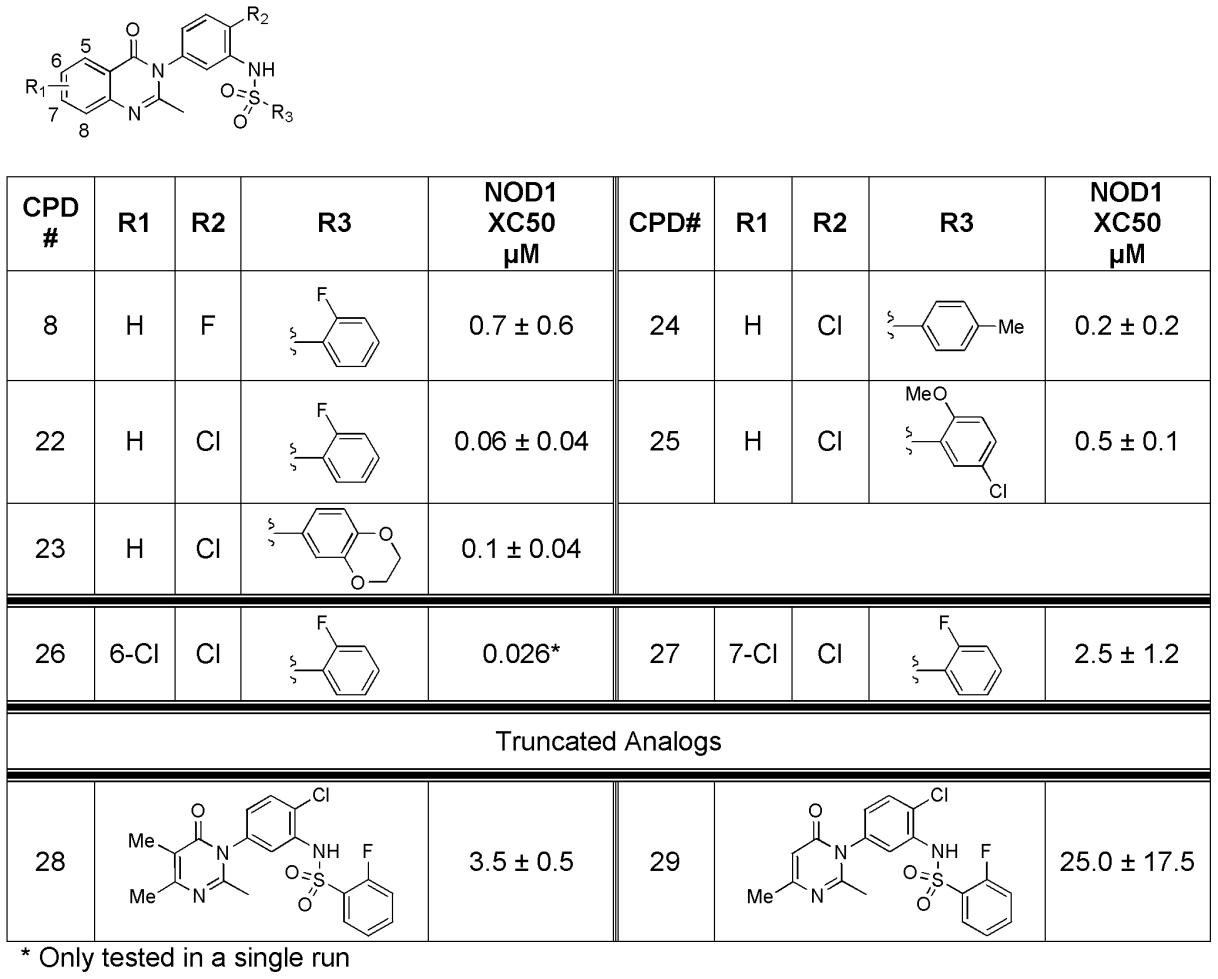
SAR for additional quinazolininone analogs (compounds 22–29) in the 293/hNOD1 assay of iE-DAP stimulated IL-8 production.

To further investigate the potential for the three series, we selected a small set from each for evaluation of their physicochemical properties, including CLND solubility, permeability, chromatographic hydrophobicity (Chromlog D at 3 pHs), polar surface area, immobilized artificial membrane (IAM) binding, human serum albumin (HSA) binding, and α1-acid glycoprotein (AGP) binding. The data for exemplar compounds including the 3 original hit compounds from each series is presented in Table S1. After the analysis, the quinazolininone biarylsulfonamide (GSK223) series stood out as having the best physicochemical properties in all aspects. The quinazolininone biarylsulfonamides (GSK223) series was thus selected for further optimization. Understanding that the removal of the fluoro at the 2-position on the core phenyl caused a significant decrease in activity (position R1 in Figure 6: compare GSK223 and compound 14), we then looked at replacement of the fluoro on the central core with a chlorine. Satisfyingly, the change resulted in >1 log boost in activity (293/hNOD1 IC_50_ for compound **8** = 0.7 µM vs compound **22** = 0.06 µM). This boost in activity held true for all analogs prepared and tested with the chloro replacement (Figure 8). Additionally, truncation of the quinazolininone to either the dimethyl or monomethyl resulted in a loss of activity (compounds **28** and **29**). Finally, while substitution at the 7-position (compound **27**) diminished NOD1 activity, 6-substitution gave a slight increase in activity (compound **26**). Overall, the quinazolininone biarylsulfonamides have very promising physicochemical properties and excellent activity in NOD1 cellular assays.

### Inverse Relationship between NOD1 Agonist Activity and Inhibitor Concentration

To better understand the mechanism of action of the NOD1 pathway inhibitors, the relationship between inhibitor compound concentration and NOD1 agonist activity was examined. The IL-8 production by HCT116 cells in response to increasing concentrations of Tri-DAP was determined in the absence and presence of different concentrations of compound. Pre-incubation of cells with increasing concentrations of both a quinazolininone (compound **22**) or an aminobenzothiazole (compound **16**) resulted in a progressive reduction in both the apparent EC_50_ and maximum response of Tri-DAP (Figure S3). These data are suggestive of a noncompetitive interaction or behavior between the NOD1 agonist and the biarylsulfonamide inhibitor compounds. However, agonist-inhibitor relationships derived from cell-based data need to be interpreted with caution, particularly when validation with a direct binding assay is unavailable.

### Inhibition of Type I Interferon Response

**Figure 9:**
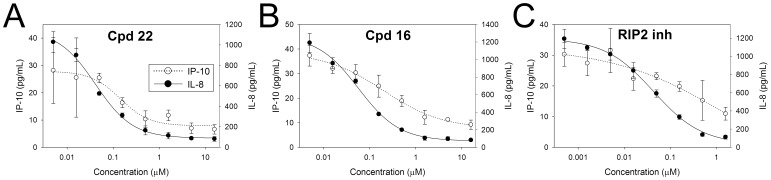
NOD1 inhibitors antagonize Tri-DAP induced type I IFN response. HT-29 colon carcinoma cells were pre-incubated with more potent analogues from the quinazolininone (compound **22**) and aminobenzothiazole (compound **16**) series (5 nM –16 µM), or with a RIP2 kinase inhibitor (0.5 nM –1.6 µM) followed by stimulation with 50 µg/mL Tri-DAP for 24 hours. IL-8 and IP-10/CXCL10 secreted into conditioned medium were measured by HTRF assay and ELISA, respectively. Results are shown as the mean ± SD of chemokine concentrations determined from triplicate treatment wells. Data are representative of two experiments.

Besides NF-κB and MAPK pathways, stimulation of NOD1 (but not NOD2) in intestinal epithelial cells has also been demonstrated to induce a TBK1/IKKε/IRF7/type I interferon pathway leading to secretion of IFN-induced proteins including IP-10/CXCL10 [10]. Activation of this pathway in gut epithelia is considered to represent an additional mechanism whereby NOD1 contributes to mucosal host defense. Therefore, we tested whether the most potent biarylsulphonamide compounds could also inhibit this NOD1 mediated type I IFN response by determining the effect on Tri-DAP stimulated IP-10 secretion in HT-29 colon carcinoma cells, which express endogenous NOD1 but not NOD2. As shown in Figure 9, both the quinazolininone compound **22** and aminobenzothiazole compound **16** dose-dependently inhibited IP-10 release. Secretion of IP-10 was also prevented by the RIP2 inhibitor, in agreement with the NOD1-induced type I IFN response in intestinal epithelial cells being RIP2-dependent [10]. The activities of the NOD1 and RIP2 compounds for inhibition of IP-10 release were comparable to that for inhibition of IL-8 measured in the same supernatants (IC_50_ compound **22** = 0.08 µM [IP-10], 0.03 µM [IL-8]; compound **16** = 0.25 µM [IP-10], 0.04 µM [IL-8]; RIP2 inhibitor = 0.06 µM [IP-10], 0.03 µM [IL-8]). Interestingly, IFNβ was undetectable in medium of untreated and Tri-DAP stimulated HT-29 cells and, moreover, Tri-DAP failed to stimulate the release of either IP-10 or IFNβ in HCT116 cells (data not shown) despite the clear NOD1-mediated NF-κB response in HCT116 cells. Prevention of the NOD1 agonist-induced type I IFN response has also been demonstrated for the NOD1 inhibitor probe ML130 using an IFN-sensitive response element reporter assay [18].

### Activity in Primary Human Monocytes and Murine BMDM

**Figure 10:**
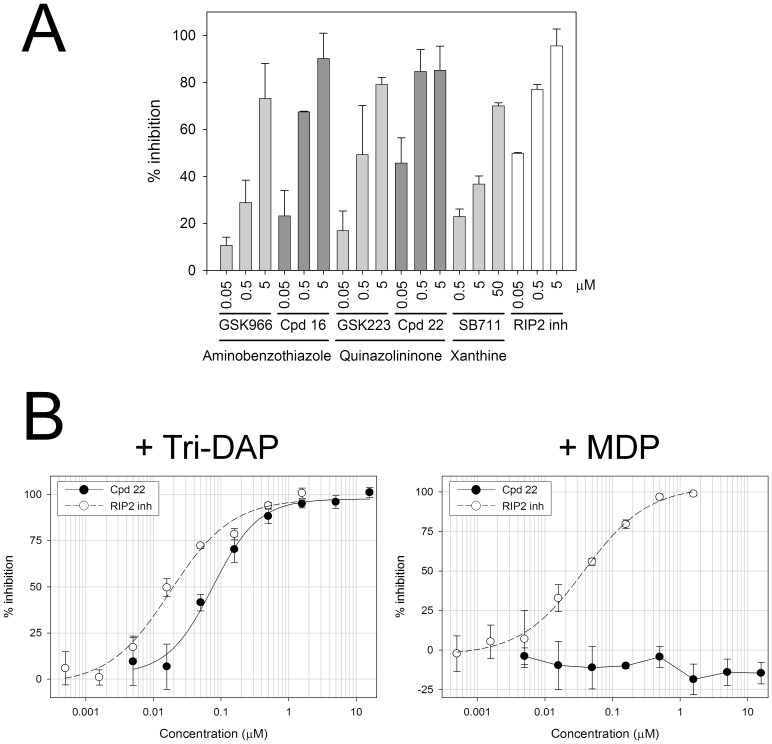
Suppression of human monocyte IL-8 secretion by selective NOD1 inhibitors. (A) Comparison of activity between original hit compounds and more potent analogues **16** and **22**. Monocytes freshly isolated from human whole blood were pre-treated with NOD1 compounds (0.05, 0.5 and 5 µM) prior to stimulation with 25 µg/mL Tri-DAP. An inhibitor of RIP2 kinase was included as a positive control. The amount of IL-8 present in the medium after 24-hour incubation was determined by HTRF assay. (B) The more potent quinazolininone compound **22** selectively inhibits Tri-DAP but not MDP stimulated IL-8 secretion. Monocytes were pre-treated with compound at the indicated concentrations for 1 hour prior to stimulation with either 25 µg/mL Tri-DAP or 0.1 µg/mL MDP for 24 hours. IL-8 release was determined by HTRF assay. The RIP2 inhibitor blocked responses to both NOD agonists, as expected, and was included as a positive control. Results are expressed as percent inhibition of stimulated IL-8 secretion. The data shown in (A) is the mean inhibition (± SD) observed across multiple experiments (n = 2 or 4) for each compound and data in (B) is the mean ± SD of triplicate treatment wells from an individual experiment repeated once with similar results.

Representative inhibitors from each of the three chemical series were also able to block NOD1-mediated IL-8 secretion in primary human monocytes. Tri-DAP (25 µg/mL) increased IL-8 secretion by these cells after 24 hours, although the magnitude of the increase was highly variable as is typical for primary monocytes isolated from different donors. Despite the variability in the NOD1 response, pre-treatment of cells with compound consistently and dose-dependently reduced Tri-DAP stimulated IL-8 secretion (Figure 10A). The quinazolininone and aminobenzothiazole analogues with improved activity in the 293/hNOD1 and HCT116 assays, (compounds **22** and **16**, Table 1, Figures 7 and 8), also gave a greater level of inhibition in monocytes compared to the corresponding original hits, GSK223 and GSK966, respectively (Figure 10A). Unfortunately the effects of the compounds on other pro-inflammatory cytokines (TNFα, IL-6 and IL-1β) could not be assessed owing to inadequate induction by Tri-DAP at the concentration used. Compound **22** did not inhibit IL-8 secretion induced by the NOD2 agonist MDP, indicating that selectivity for the NOD1 signaling pathway – at least for the quinazolininone series – is preserved in primary cells (Figure 10B). The quinazolininone series has also been demonstrated to selectively inhibit the NOD1 mediated inflammatory response in primary endothelial cells [15]. Specifically, compound **22** (referred to as GSK217 in reference [15]) inhibited IL-8 secretion from human lung microvascular endothelial cells stimulated with 10 µg/mL iE-DAP while, in contrast, the compound had no inhibitory effect on IL-8 release from cells stimulated with lipopolysaccharide (LPS, a TLR4 agonist). Based on this and other findings it has been suggested that NOD1 contributes to the endothelium’s ability to directly respond to pathogens during sepsis and acute lung injury, in addition to immune cells such as macrophages [15], [16].

To examine whether the compounds also inhibited murine NOD1 signaling, the most active quinazolininone and aminobenzothiazole analogs (compounds **22** and **16**) were also tested for their ability to inhibit NOD1 stimulated production of the chemokine KC (CXCL1) in primary murine bone marrow derived macrophages. In these experiments NOD1 was activated with the synthetic tetra-DAP peptide FK156 as well as Tri-DAP because the former ligand was found to produce a greater induction of KC, in agreement with published reports demonstrating stronger NOD1 activation by tetra-DAP rather than tri-DAP peptides in murine compared to human cells [20]. However, while the RIP2 inhibitor completely prevented FK156 or Tri-DAP-stimulated KC production by murine BMDM, compound **22** exhibited little or no inhibitory activity while compound **16** showed partial inhibition at best, irrespective of the stimulus (Figure S4). Moreover, oral administration of compound **22** in mice failed to reduce serum KC and IP-10 (CXCL10) following intraperitoneal injection of FK156 in a model of acute peritonitis despite sufficient systemic exposure to the compound (data not shown). Taken together, these findings suggest that the loss of activity for the quinazolininone and aminobenzothiazole NOD1 compound series in mice is most likely the result of species differences in NOD1 activation and signaling between rodents and humans rather than ligand-specific differences in downstream processes. We speculate that the known differences in ligand recognition between human and mouse NOD1 mapped to residues within the leucine-rich repeat domains could underlie the observed differential activity of the compounds on human versus mouse NOD1 signaling [21].

### Comparison with Previously Reported Inhibitors of NOD1 Signaling

Two chemical series of NOD1 pathway inhibitors have previously been reported, the purine-2,6-diones (ML146) and 2-aminobenzimidazole derivatives (probe ML130) [17], [18]. These series were identified using a cell-based screen of Tri-DAP stimulated NF-κB reporter activity dependent upon endogenous NOD1 in HEK293T cells, and both series were shown to be selective against NOD2 and TNFR1 stimulated pathways (MDP and TNFα stimulated IL-8 release). While the xanthine core series (SB711) we identified is clearly similar to ML146, the 2-aminobenzimidazole derivatives (ML130) also share some structural similarity to the quinazolininone and aminobenzothiazole series since all three are biarylsulfonamides. However, while the reported activity of ML130 in the NOD1-stimulated NF-κB reporter assay is slightly higher than that of ML146 (IC_50_ = 0.56 µM [17] vs 1.5 µM [Magnuson et al, 2010, URL provided above]) and comparable to several of the quinazolininone and aminobenzothiazole biarylsulfonamide compounds that we identified through SAR optimization, the most active quinazolininone analog is significantly greater again (compound **22** IC_50_ = 0.06 µM). Together with the greater inhibitory activity of certain quinazolininones, we have more fully characterized each of our series compared to the previously reported compounds by demonstrating inhibition of additional NOD1-dependent signaling pathways (NF-κB, IRF/type I IFN and MAPK) as well as selectivity over other inflammatory pathways (NOD2, TNFR1 and TLR2).

### Significance and Potential Utility of NOD1 Pathway Inhibitors

Although each of the chemical series we identified selectively inhibits NOD1-mediated signaling and pro-inflammatory cytokine production without affecting downstream signaling induced by the activation of NOD2, TLR2 or TNFR1, we do not know their definitive mechanism of action. Most importantly, it is unknown whether these compounds interact directly with NOD1. The fact that the biarylsulfonamide compounds blocked NF-κB, MAPK and type I IFN signaling pathways following NOD1 activation but do not inhibit the activity of the downstream effector kinase RIP2, demonstrates that these molecules act upstream of RIP2 and of the divergence points in these pathways. It is possible that the compounds interfere with the interaction between NOD1 and its agonist DAP, and the apparent noncompetitive interaction between NOD1 agonist and biarylsulfonamide compounds as well as the differential activity of the biarylsulfonamide compounds between human and mouse NOD1 tends to support this notion. Alternatively, the compounds could act by preventing nucleotide binding to NOD1 which is required for oligomerization and downstream signaling by NOD1 (and NOD2), as demonstrated by the inactivity of P-loop mutants [22]. However the latter mechanism is considered unlikely because both NOD1 and NOD2 preferentially bind ATP over other nucleotides with high affinity (NOD1 IC_50_ ∼15 nM [9], NOD2 IC_50_ ∼60 nM [23]) and only inhibitors with similarly high affinity would be able to effectively compete off intracellular ATP. Despite advances in purification of NOD proteins reported in the literature [8], our own attempts and that of an external collaborator, to obtain full-length NOD1 and other NLRs have been unsuccessful. However, we are continuing to work to identify the molecular target(s) of these compounds.

The selectivity of these newly identified compounds for inhibition of the NOD1 signaling pathway without influencing signaling through NOD2, TLR2 or the TNF receptor, suggests they should prove valuable chemical tools to dissect the role of NOD1 in various in vitro and organ culture models of inflammation. It is hoped that these compounds will not only aid further understanding of the role of NOD1 in initiating immune responses at mucosal surfaces, but also help to elucidate this innate immune sensor’s emerging wider role in the inflammation associated with ischemia/reperfusion injury and neutrophil influx [24], [25], diet-induced insulin resistance in adipose, liver and skeletal muscle [14], and vascular lesions of coronary arteritis [15], [16] and atherosclerosis [26]. If used in conjunction with our recently identified selective NOD2 pathway inhibitors [19], then it should be possible to determine the relative contribution of these two closely related cytosolic PRRs to the inflammatory process under investigation.

## Supporting Information

Figure S1 **Kinome plot showing the kinase inhibitory activity for original hit from each chemical series.** Approximately 300 different kinases were screened with each compound at 1 µM. Percent inhibition of kinase activity is color-coded for each individual kinase as indicated. A = SB711, B = GSK966, C = GSK223.
(TIF)

Figure S2 **Inhibition of NF-κB and MAPK signaling by more active quinazolininone and aminobenzothiazole compounds identified through SAR.** Serum-starved 293/hNOD1 cells were pre-incubated with SB711, the quinazolininone compound 9, or aminobenzothiazole compound 16 (at 1–30 µM) and then stimulated for 1 hour with 50 µg/mL Tri-DAP. The levels of total IκBα and of total and phosphorylated p38, JNK and ERK1/2 were determined by immunoblotting of whole cell lysates. Results shown are representative of two separate experiments.
(TIF)

Figure S3 **Inverse relationship between NOD1 inhibitor concentration and Tri-DAP stimulatory activity.** HCT116 cells were pre-incubated for 1 hour with or without compound at various concentrations ranging from 0.005–1 µM and then, for each compound concentration, stimulated with a concentration range of Tri-DAP (0.6–75 µg/mL). After 24 hours the amount of IL-8 secreted in to the medium was determined. (A) Tri-DAP dose-dependently increased IL-8 release with maximal stimulation at 50 µg/mL Tri-DAP and above, and an EC_50_ = 12 µg/mL (30 nmol/mL) indicated by the dotted line. (B and C) Increasing concentrations of aminobenzothiazole compound **16** (B) and quinazolininone compound **22** (C) progressively decreased the stimulatory response to Tri-DAP.
(TIF)

Figure S4 **Activity of quinazolininone and aminobenzothiazole compounds in NOD1 stimulated murine BMDM.** Murine BMDM were pretreated with compound **16** or compound **22** (0.05–16 µM) for 1 hour and then stimulated for 24 hours with 25 µg/mL of either Tri-DAP (A) or the synthetic tetra-DAP NOD1 agonist FK156 (B). An inhibitor of RIP2 kinase (0.005–5 µM) was included as a positive control. The concentration of murine chemokine KC (CXCL1) secreted into medium was determined by MSD assay. Data are the mean percent inhibition (± SD) determined from three independent experiments using cells obtained from different donor animals.
(TIF)

Table S1 **Physicochemical properties of exemplar compounds from each chemical series.** Parameters measured include CLND solubility (Solubility determination by precipitation of 10 mM DMSO stock concentration to 5% DMSO pH7.4 phosphate buffered saline with quantification by Chemiluminescent Nitrogen Detection); chromatographic hydrophobicity index (Chrom log D, a measure of lipophilicity); permeability across an artificial membrane; polar surface area (PSA); human serum albumin (HSA) binding; α1-acid glycoprotein (AGP) binding; immobilized artificial membrane (IAM) binding. ND = not determined.
(DOCX)

Methods S1 **Chemical syntheses, or structural confirmation of purchased compounds, for all compounds listed in tables (compounds 1–29).**
(DOC)
