# Supplementary figures and images for: Identification of Selective Small Molecule Inhibitors of the Nucleotide-Binding Oligomerization Domain 1 (NOD1) Signaling Pathway
Source: PLoS One. 2014 May 7;9(5):e96737. doi: 10.1371/journal.pone.0096737 (PMC4013053; doi:10.1371/journal.pone.0096737)

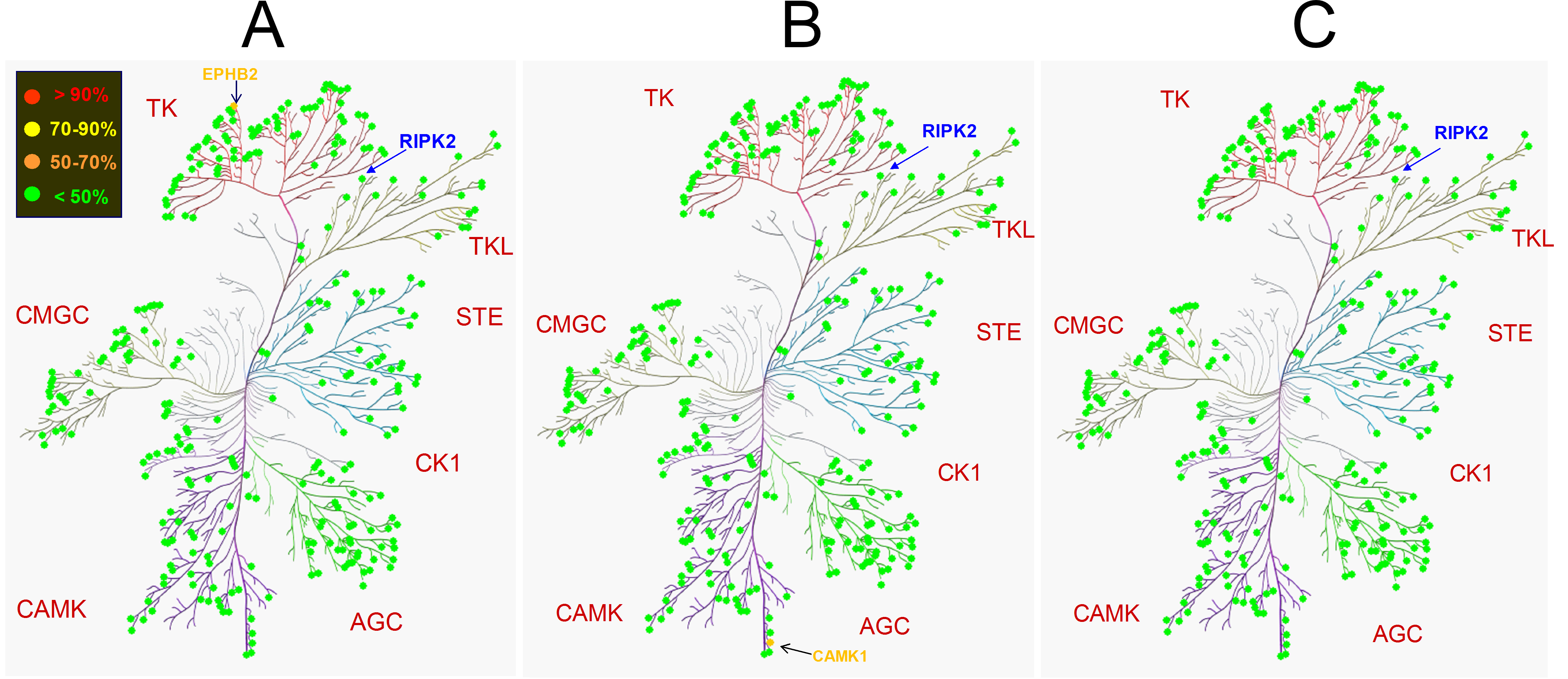

Supplement: Figure S1 — Kinome plot showing the kinase inhibitory activity for original hit from each chemical series. Approximately 300 different kinases were screened with each compound at 1 µM. Percent inhibition of kinase activity is color-coded for each individual kinase as indicated. A = SB711, B = GSK966, C = GSK223. (TIF) [file pone.0096737.s001.tif]

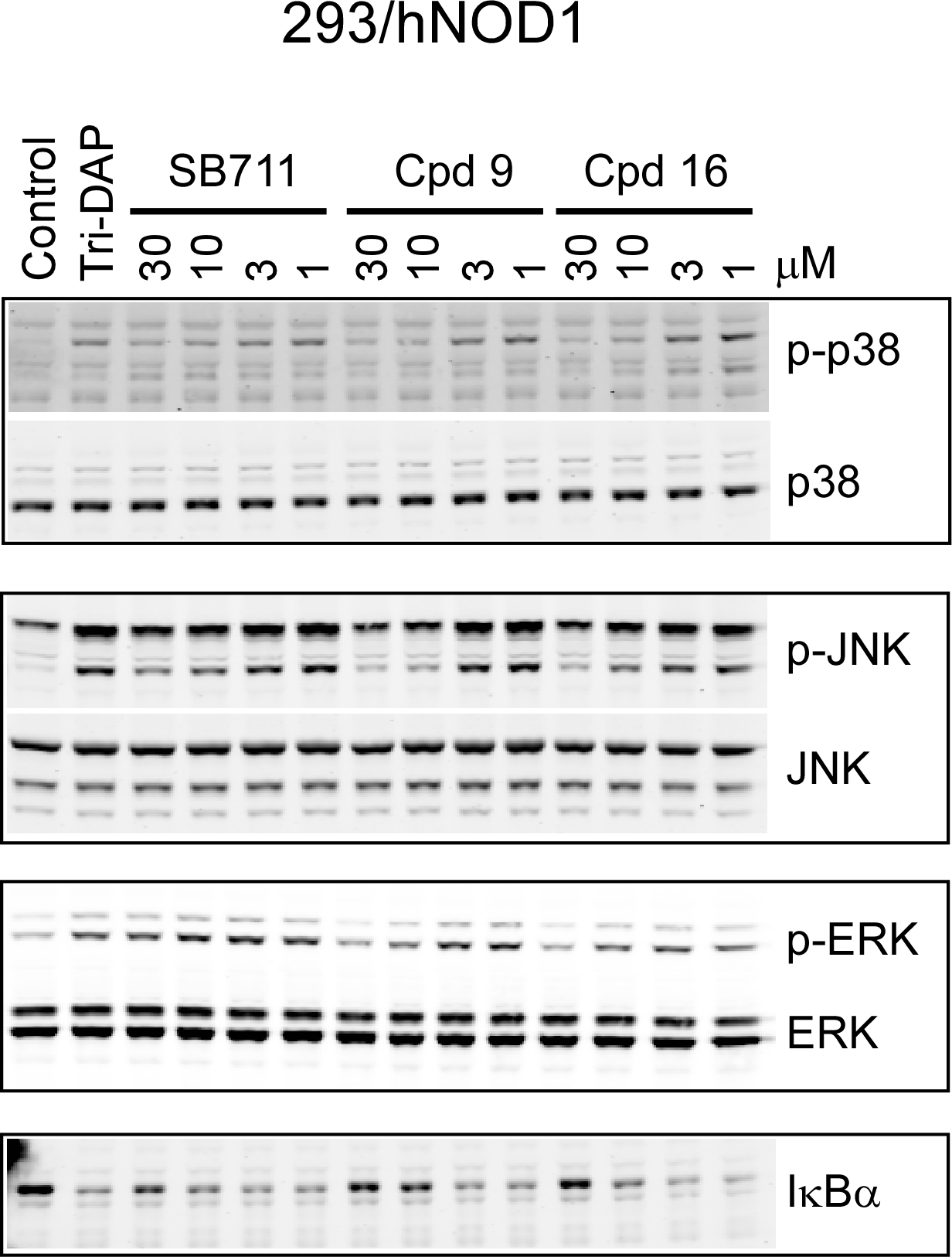

Supplement: Figure S2 — Inhibition of NF-κB and MAPK signaling by more active quinazolininone and aminobenzothiazole compounds identified through SAR. Serum-starved 293/hNOD1 cells were pre-incubated with SB711, the quinazolininone compound 9, or aminobenzothiazole compound 16 (at 1–30 µM) and then stimulated for 1 hour with 50 µg/mL Tri-DAP. The levels of total IκBα and of total and phosphorylated p38, JNK and ERK1/2 were determined by immunoblotting of whole cell lysates. Results shown are representative of two separate experiments. (TIF) [file pone.0096737.s002.tif]

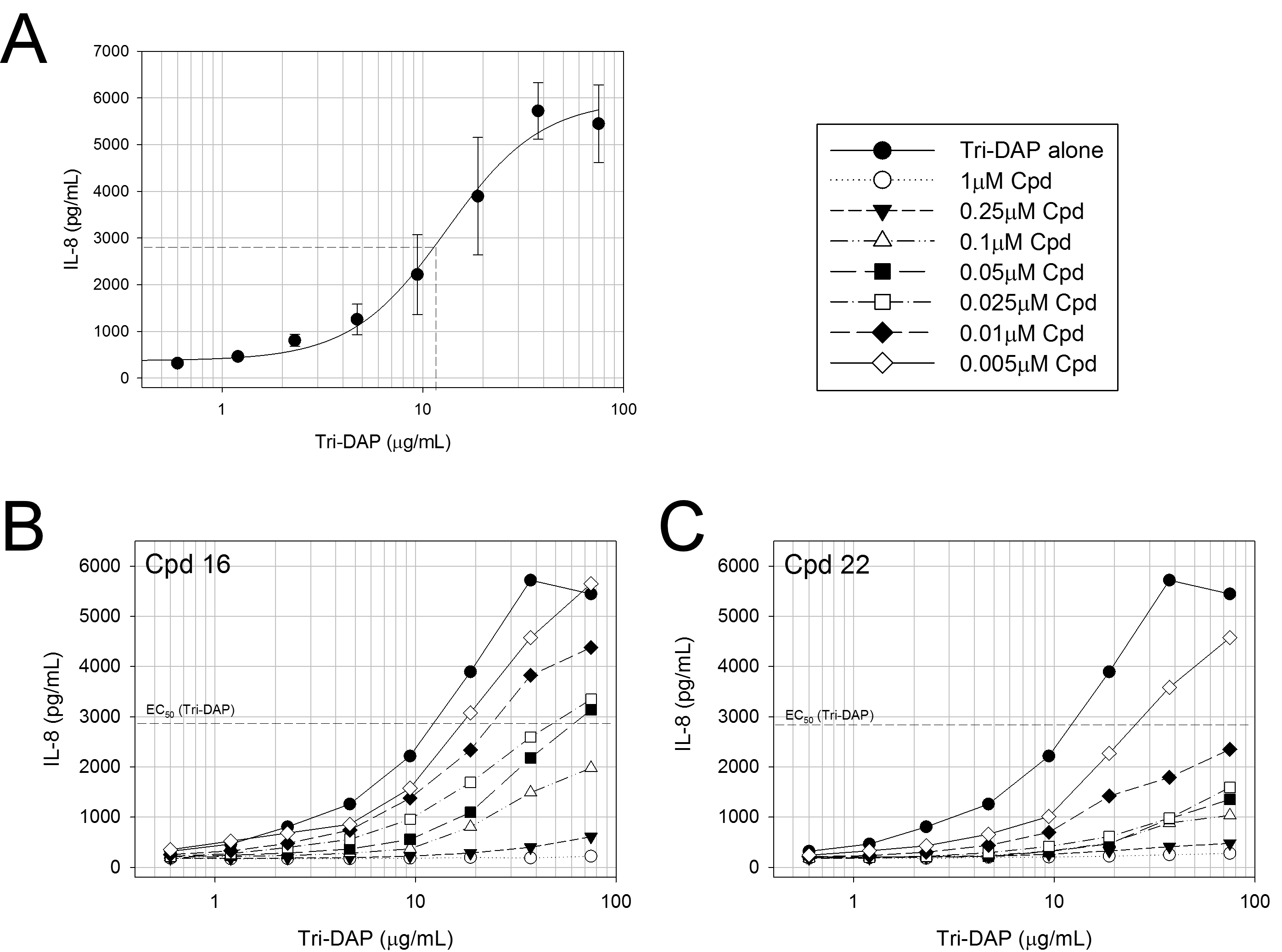

Supplement: Figure S3 — Inverse relationship between NOD1 inhibitor concentration and Tri-DAP stimulatory activity. HCT116 cells were pre-incubated for 1 hour with or without compound at various concentrations ranging from 0.005–1 µM and then, for each compound concentration, stimulated with a concentration range of Tri-DAP (0.6–75 µg/mL). After 24 hours the amount of IL-8 secreted in to the medium was determined. (A) Tri-DAP dose-dependently increased IL-8 release with maximal stimulation at 50 µg/mL Tri-DAP and above, and an EC50 = 12 µg/mL (30 nmol/mL) indicated by the dotted line. (B and C) Increasing concentrations of aminobenzothiazole compound 16 (B) and quinazolininone compound 22 (C) progressively decreased the stimulatory response to Tri-DAP. (TIF) [file pone.0096737.s003.tif]

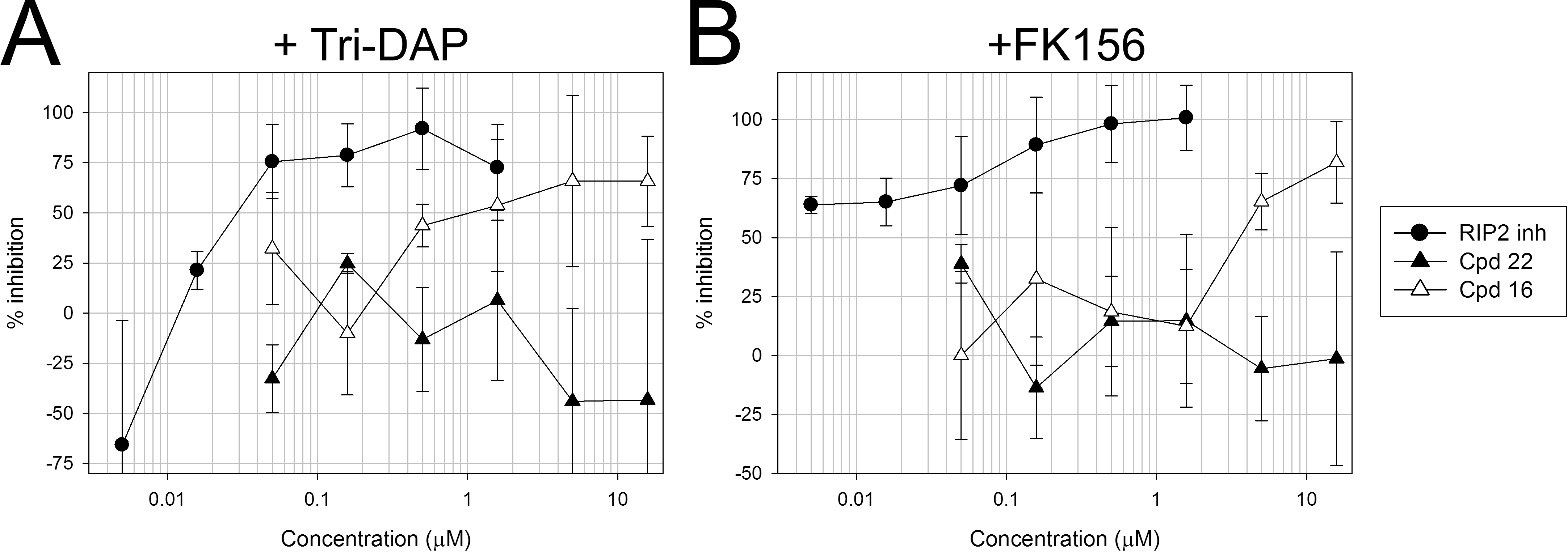

Supplement: Figure S4 — Activity of quinazolininone and aminobenzothiazole compounds in NOD1 stimulated murine BMDM. Murine BMDM were pretreated with compound 16 or compound 22 (0.05–16 µM) for 1 hour and then stimulated for 24 hours with 25 µg/mL of either Tri-DAP (A) or the synthetic tetra-DAP NOD1 agonist FK156 (B). An inhibitor of RIP2 kinase (0.005–5 µM) was included as a positive control. The concentration of murine chemokine KC (CXCL1) secreted into medium was determined by MSD assay. Data are the mean percent inhibition (± SD) determined from three independent experiments using cells obtained from different donor animals. (TIF) [file pone.0096737.s004.tif]
