# Supplementary material for: Identification of Selective Small Molecule Inhibitors of the Nucleotide-Binding Oligomerization Domain 1 (NOD1) Signaling Pathway
Source: PLoS One. 2014 May 7;9(5):e96737. doi: 10.1371/journal.pone.0096737 (PMC4013053; doi:10.1371/journal.pone.0096737)
Supplement: Table S1 — Physicochemical properties of exemplar compounds from each chemical series. Parameters measured include CLND solubility (Solubility determination by precipitation of 10 mM DMSO stock concentration to 5% DMSO pH7.4 phosphate buffered saline with quantification by Chemiluminescent Nitrogen Detection); chromatographic hydrophobicity index (Chrom log D, a measure of lipophilicity); permeability across an artificial membrane; polar surface area (PSA); human serum albumin (HSA) binding; α1-acid glycoprotein (AGP) binding; immobilized artificial membrane (IAM) binding. ND = not determined. (DOCX) [file pone.0096737.s005.docx]

**Table S1 Physico-chemical properties of exemplar compounds from each chemical series**

| **Parameter** | **Xanthine** | | **Quinazolininone** | | | **Aminobenzothiazole** | | |
| --- | --- | --- | --- | --- | --- | --- | --- | --- |
|  | SB711 | GW543 | GSK223 | Cpd 8 | Cpd 13 | GSK966 | Cpd 16 | Cpd 21 |
| CLND solubility (μM) | 2 | 4 | 327 | 391 | 423 | 41 | 14 | 46 |
| Chrom LogD pH 7.4 | 5.95 | 7.38 | 4.27 | 3.24 | 2.87 | 4.64 | 4.89 | 5.17 |
| Permeability (nm/s) | ND | 370 | 490 | 350 | 260 | 300 | 220 | 230 |
| PSA (Å) | 73 | 73 | 81 | 81 | 81 | 88 | 88 | 93 |
| HSA binding (%) | 95.94 | 96.82 | 97.38 | 96.05 | 96.08 | 97.14 | 97.2 | 98.01 |
| AGP binding (%) | ND | 90.69 | 80.81 | 80.38 | 81.23 | 83.38 | 85.71 | 87.41 |
| IAM binding (log K) | 2.41 | 2.67 | 1.68 | 1.42 | 1.36 | 2.38 | 2.45 | 2.40 |

Parameters measured include CLND solubility (Solubility determination by precipitation of 10mM DMSO stock concentration to 5% DMSO pH7.4 phosphate buffered saline with quantification by Chemiluminescent Nitrogen Detection); chromatographic hydrophobicity index (Chrom log D, a measure of lipophilicity); permeability across an artificial membrane; polar surface area (PSA); human serum albumin (HSA) binding; α1-acid glycoprotein (AGP) binding; immobilized artificial membrane (IAM) binding. ND = not determined.
