# Supplementary material for: Identification of Selective Small Molecule Inhibitors of the Nucleotide-Binding Oligomerization Domain 1 (NOD1) Signaling Pathway
Source: PLoS One. 2014 May 7;9(5):e96737. doi: 10.1371/journal.pone.0096737 (PMC4013053; doi:10.1371/journal.pone.0096737)
Supplement: Methods S1 — Chemical syntheses, or structural confirmation of purchased compounds, for all compounds listed in tables (compounds 1–29). (DOC) [file pone.0096737.s006.doc]

**[Rickard et al, Identification of selective NOD1 pathway inhibitors]**

**Methods S1**

High resolution mass spectrometry for collection compounds by LTQ-Orbitrap Discovery MS (Thermo Scientific) operating in electrospray ionization, positive mode (ESI+). Resolving power 15,000-30,000. System was calibrated using Sodium TFA solution at start of day. Sample introduced by LC using an Agilent 1200 series LC stack. Data collected and processed with Xcalibur software, version 2.0.7.

| **Compound No.** | **Molecular Formula** | **Calculated Exact Mass** | **Observed Mass (ESI M+H)** |
| --- | --- | --- | --- |
| 1 | C21H20N4O2S | 392.1307 | 393.1381 |
| GSK711 | C21H20N4O2S | 392.1307 | 393.1378 |
| 2 | C22H22N4O2S | 406.1463 | 407.1536 |
| 3 | C19H24N4O2S | 372.16 | NA |
| 4 | C22H22N4O2S | 406.1463 | 407.1538 |
| 5 | C21H20N4O2S | 392.1307 | 393.1380 |
| 6 | C18H20N4O2S | 356.1307 | 357.1381 |
| 7 | C20H18N4O2S | 378.1150 | 379.1224 |
| 8 | C21H15F2N3O3S | 427.0802 | 428.0874 |
| 9 | C19H13ClFN3O3S2 | 449.0071 | 450.0144 |
| GSK223 | C21H18FN3O3S2 | 443.0774 | 444.0847 |
| 10 | C21H14F3N3O3S | 445.0708 | 446.0781 |
| 11 | C23H20FN3O3S | 437.1209 | 438.1283 |
| 12 | C21H14Cl2FN3O3S | 477.0117 | 478.0191 |
| 13 | C19H14FN3O3S2 | 415.0461 | 416.0533 |
| 14 | C21H19N3O3S2 | 425.0868 | 426.0941 |
| 15 | C21H16N4O5S2 | 468.0562 | 469.0635 |
| 16 | C22H19N3O3S2 | 437.0868 | 438.0942 |
| 17 | C22H18FN3O3S2 | 455.0774 | 456.0847 |
| 18 | C23H20N4O4S2 | 480.0926 | 481.1000 |
| 19 | C23H15ClN4O3S2 | 494.0274 | 495.0347 |
| 20 | C22H19N3O4S2 | 453.0817 | 454.0889 |
| GSK966 | C21H16FN3O3S2 | 441.0617 | 442.0680 |
| 21 | C20H15ClN2O5S | 430.0390 | 430.0384 |
| 22 | C21H15ClFN3O3S | 443.0507 | 444.0581 |
| 23 | C23H18ClN3O5S | 483.0656 | 484.0727 |
| 24 | C22H18ClN3O3S | 439.0757 | 440.0832 |
| 25 | C22H17Cl2N3O4S | 489.0317 | 490.0393 |

Experimental write up of compound 26: *N*-(2-chloro-5-(6-chloro-2-methyl-4-oxoquinazolin-3(4H)-yl)phenyl)-2-fluorobenzenesulfonamide

Step-1: 6-chloro-2-methyl-4H-benzo[d][1,3]oxazin-4-one

A mixture o of 2-amino-5-chlorobenzoic acid (1 g, 5.83 mmol) and acetic anhydride (3.30 ml, 35.0 mmol) was heated at 130 °C for 2 hr. After cooling to room temperature, an excess amount of acetic anhydride was removed under reduced pressure at 40 °C. The residual solid was suspended in hexane with stirring, collected by filtration and dried in vacuo to afford 6-chloro-2-methyl-4H-benzo[d][1,3]oxazin-4-one (520 mg, 1.46 mmol, 25.1 % yield). MS (m/z) 196 (M+H^+^).

Step-2: 3-(3-amino-4-chlorophenyl)-6-chloro-2-methylquinazolin-4(3H)-one

To a stirred solution of 6-chloro-2-methyl-4H-benzo[d][1,3]oxazin-4-one (520 mg, 2.66 mmol) in pyridine (5 mL) was added 4-chlorobenzene-1,3-diamine (455 mg, 3.19 mmol). The reaction mixture was stirred at 110 °C for 6 hr. TLC indicated completion of the reaction. The reaction mixture was cooled to rt and distilled under vacuo to acquire crude product. The crude product was added to a silica gel(100-200 mesh) column and was eluted with EtOAc; The fractions were collected and concentrated under vacuo to get 3-(3-amino-4-chlorophenyl)-6-chloro-2-methylquinazolin-4(3H)-one (450 mg, 0.773 mmol, 29.1 % yield). MS (m/z) 320 (M+H^+^).

Step-3: N-(2-chloro-5-(6-chloro-2-methyl-4-oxoquinazolin-3(4H)-yl)phenyl)-2-fluorobenzenesulfonamide

To a solution of 3-(3-amino-4-chlorophenyl)-6-chloro-2-methylquinazolin-4(3H)-one (150 mg, 0.468 mmol) in dichloromethane (DCM) (5 mL) was added pyridine (0.076 mL, 0.937 mmol) at RT and the contents were stirred for 20min and then 2-fluorobenzene-1-sulfonyl chloride (109 mg, 0.562 mmol) was added. The reaction mixture was stirred at RT for 72h. The reaction was monitored by LCMS; it showed 50% of desired mass peak. The reaction mixture was evaporated under reduced pressure to remove the solvents. The crude material was purified by preparative HPLC to afford N-(2-chloro-5-(6-chloro-2-methyl-4-oxoquinazolin-3(4H)-yl)phenyl)-2-fluorobenzenesulfonamide (110 mg, 0.226 mmol, 48.3% yield) as an off-white solid. MS (m/z) 476, 478, 480 (M-H^+^); ^1^H NMR (400 MHz, DMSO-*d*_6_) δ ppm 10.6 (s, 1 H), 8.05 (t, 1 H), 7.85 (dd, 1 H), 7.68 (m, 3 H), 7.61 (d, 1 H), 7.52 (m, 1 H), 7.35 (m, 3 H), 2.05 (s, 3 H).

Experimental write up of 27: N-(2-chloro-5-(7-chloro-2-methyl-4-oxoquinazolin-3(4H)-yl)phenyl)-2-fluorobenzenesulfonamide

Step-1: 7-chloro-2-methyl-4H-benzo[d][1,3]oxazin-4-one

A mixture o of 2-amino-4-chlorobenzoic acid (1.00 g, 5.83 mmol) and acetic anhydride (3.30 ml, 35.0 mmol) was heated at 130 °C for 2 hr. After being cooled to room temperature, excess amount of acetic anhydride was removed under reduced pressure at 40 °C. The residual solid was suspended in hexane with stirring, collected by filtration and dried in vacuo to afford 7-chloro-2-methyl-4H-benzo[d][1,3]oxazin-4-one (520 mg, 2.58 mmol, 44.2 % yield). MS (m/z) 194, 196 (M+H^+^).

Step-2: 3-(3-amino-4-chlorophenyl)-7-chloro-2-methylquinazolin-4(3H)-one

To a stirred solution of 7-chloro-2-methyl-4H-benzo[d][1,3]oxazin-4-one (550 mg, 2.81 mmol) in pyridine (5 mL) was added 4-chlorobenzene-1,3-diamine (481 mg, 3.37 mmol). The reaction mixture was stirred at 110 °C for 6 hr. TLC indicated completion of the reaction. The reaction mixture was cooled to rt and distilled under vacuum. The crude product was added to a silica gel (100-200 mesh) column and was eluted with EtOAc; Collected fractions and concentrated in vacuo to afford 3-(3-amino-4-chlorophenyl)-7-chloro-2-methylquinazolin-4(3H)-one (200 mg, 0.625 mmol, 22.22 % yield).

Step-3: *N*-(2-chloro-5-(7-chloro-2-methyl-4-oxoquinazolin-3(4H)-yl)phenyl)-2-fluorobenzenesulfonamide

To a stirred solution of3-(3-amino-4-chlorophenyl)-7-chloroquinazolin-4(3H)-one (200 mg, 0.653 mmol) in dichloromethane (DCM) (10 mL) was added 2-fluorobenzene-1-sulfonyl chloride (127 mg, 0.653 mmol) and pyridine (0.053 mL, 0.653 mmol) . The reaction mixture was stirred at rt for 48hr. The reaction mixture was diluted with EtOAc (20 mL) and water (10 mL) and the layers separated. The aqueous layer was back extracted with EtOAc (10 mL). The combined organic layers were dried over Na_2_SO_4_, filtered and concentrated in vacuo. The crude product was added to a silica gel (100-200 mesh) column and was eluted with Hex/EtOAc (30/70). The fractions were collected and concentrated in vacuo to afford N-(2-chloro-5-(7-chloro-2-methyl-4-oxoquinazolin-3(4H)-yl)phenyl)-2-fluorobenzenesulfonamide (110 mg, 0.210 mmol, 32.2 % yield). MS (m/z) 478, 480 (M+H^+^); ^1^H NMR (400 MHz, DMSO-*d*_6_) δ ppm 10.6 (s, 1 H), 8.09 (d, 1 H), 7.70 (m, 3 H), 7.60 (dd, 1 H), 7.53 (m, 2 H), 7.38 (m, 3 H), 2.05 (s, 3 H).

Experimental write up of 28: *N*-(2-chloro-5-(2,4,5-trimethyl-6-oxopyrimidin-1(6H)-yl)phenyl)-2-fluorobenzenesulfonamide

Step-1: Preparation of (Z)-ethyl 3-acetamido-2-methylbut-2-enoate

To a solution of ethyl 2-methyl-3-oxobutanoate (1.50 g, 10.40 mmol) in toluene (85 mL) was added acetamide (3.07 g, 52.0 mmol) and tosic acid (0.099 g, 0.520 mmol) at RT. The flask was fitted with dean stark trap, and the mixture was refluxed at 130 oC for 16h. The reaction was monitored by TLC. When complete, the reaction was diluted with diethyl ether and washed with water (3 x 50 mL). The combined aqueous layers were washed with diethyl ether (2 x 30 mL), and the combined organic layers were dried over sodium sulfate and concentrated. The crude material was added to a silica gel column and was eluted with Hex/EtOAc (9:1); The fractions were collected and concentrated to get (Z)-ethyl 3-acetamido-2-methylbut-2-enoate (900 mg, 4.86 mmol, 46.7 % yield). ^1^H NMR (400 MHz, CDCl_3_) δ ppm 11.61 (br. s, 1 H), 4.19 (q, 2 H), 2.40 (s, 3 H), 2.15 (s, 3 H), 1.81 (s, 3 H), 1.29 (m, 3 H).

Step-2: 3-(3-amino-4-chlorophenyl)-2,5,6-trimethylpyrimidin-4(3H)-one

To a solution of 4-chlorobenzene-1,3-diamine (831 mg, 5.83 mmol) in toluene (50 mL) added trimethylaluminium (9.72 mL, 19.44 mmol) at 10 oC. The reaction mixture was stirred for 20 min under nitrogen, and (Z)-ethyl 3-acetamido-2-methylbut-2-enoate was added (900 mg, 4.86 mmol) followed by stirring at 100 oC for 10h. The reaction was monitored by TLC. When complete, the reaction was quenched with ammonium chloride solution (100mL), and the layers were separated. The aqeous layer was extracted with EtOAc (3 x 80 mL). The combined organic layers were washed with sodium bicarbonate solution (2 x 80 mL), and the organic layer was dried over sodium sulfate. The crude product was added to a silica gel (100-200mesh) column and was eluted with CH_2_Cl_2_/MeOH (99:1). The fractions were collected and concentrated to afford 3-(3-amino-4-chlorophenyl)-2,5,6-trimethylpyrimidin-4(3H)-one (140 mg, 0.478 mmol, 9.83 % yield) and 3-(5-amino-2-chlorophenyl)-2,5,6-trimethylpyrimidin-4(3H)-one (130 mg, 0.493 mmol, 10.14 % yield). The regioselectivity was confirmed by NOESY. MS (m/z) 263.9 (M+H^+^).

Step-3: N-(2-chloro-5-(2,4,5-trimethyl-6-oxopyrimidin-1(6H)-yl)phenyl)-2-fluorobenzenesulfonamide

To a solution of 3-(3-amino-4-chlorophenyl)-2,5,6-trimethylpyrimidin-4(3H)-one (120 mg, 0.455 mmol) in Dichloromethane (DCM) (10 mL) was added pyridine (0.074 mL, 0.910 mmol) at RT and the contents were stirred at RT for 20min and then 2-fluorobenzene-1-sulfonyl chloride (106 mg, 0.546 mmol) was added and the reaction mixture was stirred at RT for 48h. The reaction was monitored by LCMS; it showed 45% of desired mass peak. The reaction mass was diluted with DCM (50mL), it was washed with water (2 x 30 mL), the organic layer was washed with 0.1M HCl (2 x 30 mL), the organic layer was dried over sodium sulfate and concentrated to get the crude. The crude product was added to a silica gel column and was eluted with CH2Cl2/MeOH (99:1). Collected fractions: concentrated to obtain N-(2-chloro-5-(2,4,5-trimethyl-6-oxopyrimidin-1(6H)-yl)phenyl)-2-fluorobenzenesulfonamide (27 mg, 0.062 mmol, 13.60 % yield) as off white powder. MS (m/z) 420, 422 (M-H^+^). ^1^H NMR (400 MHz, DMSO-*d*_6_) δ ppm 10.6 (s, 1 H), 7.70 (m, 2 H), 7.58 (d, 1 H), 7.41 (t, 1 H), 7.31 (m, 2 H), 7.25 (d, 1 H), 2.21 (s, 3H), 1.95 (s, 6H).

Experimental write up of 29: : N-(2-chloro-5-(2,4-dimethyl-6-oxopyrimidin-1(6H)-yl)phenyl)-2-fluorobenzenesulfonamide

Step-1: (Z)-ethyl 3-aminobut-2-enoate

To a solution of ethyl 3-oxobutanoate (1. g, 7.68 mmol) in methanol (100 mL) was added ammonium acetate (2.96 g, 38.4 mmol), and the contents were stirred at RT for 48h. The reaction was monitored by TLC. When complete, the reaction was concentrated to remove the methanol. It was diluted with DCM (100mL) and washed with water (3 x 200 mL). The combined aqueous layers were extracted with EtOAc (2 x 200 mL); the combined organic layers were dried over sodium sulfate and concentrated to afford crude (Z)-ethyl 3-aminobut-2-enoate (900 mg, 6.72 mmol, 87 % yield). MS (m/z) 130 (M+H^+^).

Step-2: (Z)-ethyl 3-acetamidobut-2-enoate

To a solution of (Z)-ethyl 3-aminobut-2-enoate (100 mg, 0.774 mmol) in THF was added Ac_2_O (0.073 mL, 0.774 mmol) and pyridine (0.063 mL, 0.774 mmol), and the contents were refluxed for 16h. The reaction was evaporated to remove THF and then dissolved in EtOAc (20mL). The organic layer was washed with water (2 x 20 mL). The combined aqueous layers were washed with EtOAc (2 x 20 mL). The combined organic layers were washed with brine solution (2 x 30 mL), the organic layer was dried over sodium sulfate and concentrated. The crude product was added to a silica gel column and was eluted with Hex/EtOAc (9:1). The fractions containing product were concentrated to afford the (Z)-ethyl 3-acetamidobut-2-enoate (60 mg, 0.350 mmol, 45.3 % yield). MS (m/z) 170 (M-H^+^).

Step-3: 3-(3-amino-4-chlorophenyl)-2,6-dimethylpyrimidin-4(3H)-one

To a solution of 4-chlorobenzene-1,3-diamine (275 mg, 1.928 mmol) in toluene (100 mL) was added trimethylaluminium (3.50 mL, 7.01 mmol) at 10 oC. The reaction was stirred for 20 min under nitrogen, then (Z)-ethyl 3-acetamidobut-2-enoate (300 mg, 1.752 mmol) was added, and the contents were stirred at 100oC for 10h. The reaction was quenched with ammonium chloride solution (80mL), and the aqueous layer was extracted with EtOAc (3 x 30 mL). The combined organic layers were washed with sodium bicarbonate solution (3 x 30 mL). The organic layer was dried over sodium sulfate and concentrated. The crude product was added to a silica gel (100-200mesh) column and was eluted with CH_2_Cl_2_/MeOH. The fractions were collected and concentrated to afford the 3-(3-amino-4-chlorophenyl)-2,6-dimethylpyrimidin-4(3H)-one (60 mg, 0.236 mmol, 13.48 % yield) and 3-(5-amino-2-chlorophenyl)-2,6-dimethylpyrimidin-4(3H)-one (60 mg, 0.234 mmol, 13.33 % yield). MS (m/z) 250 (M-H^+^). Regioselectivity was confirmed by NOESY.

Step-4: N-(2-chloro-5-(2,4-dimethyl-6-oxopyrimidin-1(6H)-yl)phenyl)-2-fluorobenzenesulfonamide

To a solution of 3-(3-amino-4-chlorophenyl)-2,6-dimethylpyrimidin-4(3H)-one (60 mg, 0.240 mmol) in dichloromethane (DCM) (10 mL) was added pyridine (0.039 mL, 0.481 mmol) at RT. The reaction was stirred at RT for 20min and then 2-fluorobenzene-1-sulfonyl chloride (56.1 mg, 0.288 mmol) was added, and the reaction mixture was stirred at RT for 48h. The reaction was monitored by LCMS; it showed 38% of desired mass peak. The reaction was diluted with DCM (50mL) and washed with water (3 x 30 mL). The organic layer was washed with 0.1M HCl (2 x 30 mL) and dried over sodium sulfate and concentrated. The crude product was added to a silica gel column and was eluted with CH_2_Cl_2_/MeOH (99:1). The pure fractions were combined and concentrated to afford *N*-(2-chloro-5-(2,4-dimethyl-6-oxopyrimidin-1(6H)-yl)phenyl)-2-fluorobenzenesulfonamide (25 mg, 0.060 mmol, 24.87 % yield) as an off white powder. MS (m/z) 406, 422 (M-H^+^). ^1^H NMR (400 MHz, DMSO-*d*_6_) δ ppm 10.6 (s, 1 H), 7.70 (m, 2 H), 7.60 (d, 1 H), 7.4e (t, 1 H), 7.30 (m, 3 H), 6.23 (s, 1 H), 2.20 (s, 3H), 1.98 (s, 3H).
